# Integrating Anti-Phosphatidylserine/Prothrombin Antibodies Testing into Antiphospholipid Syndrome Diagnostics: A Multidomain, Expert Perception-Based Health Technology Assessment

**DOI:** 10.3390/diagnostics16030434

**Published:** 2026-02-01

**Authors:** Michele Cioffi, Valentina Oddone, Massimo Radin, Irene Cecchi, Alice Barinotti, Silvia Grazietta Foddai, Nicola Di Gaetano, Nicoletta Pagani, Andrea Colmegna, Simone Baldovino, Roberta Fenoglio, Dario Roccatello, Savino Sciascia

**Affiliations:** 1 University Center of Excellence on Nephrologic, Rheumatologic and Rare Diseases (ERK-Net, ERN-Reconnect and RITA-ERN Member) with Nephrology and Dialysis Unit and Center of Immuno-Rheumatology and Rare Diseases (CMID), Coordinating Center of the Interregional Network for Rare Diseases of Piedmont and Aosta Valley, ASL Città di Torino, San Giovanni Bosco Hub Hospital, 10154 Turin, Italy; 2 Department of Clinical and Biological Sciences, University of Turin, 10125 Turin, Italy; 3 Werfen, 08908 Barcelona, Spainnpagani@werfen.com (N.P.);

**Keywords:** health technology assessment, anti-prothrombin antibodies, antiphospholipid syndrome, antiphospholipid antibodies

## Abstract

**Background**: Antiphospholipid syndrome (APS) is diagnosed by characteristic clinical manifestations supported by positivity for lupus anticoagulant, anticardiolipin, and anti-β2-glycoprotein I antibodies. However, a proportion of patients, especially those with systemic lupus erythematosus, remain seronegative despite high clinical suspicion. Anti-phosphatidylserine/prothrombin antibodies (aPS/PT) have emerged as potential biomarkers in this setting. We conducted an *expert perception-based* Health Technology Assessment (HTA) to evaluate the clinical, ethical, and organizational impact of implementing aPS/PT testing. **Methods**: A structured HTA was performed across five domains: safety, perceived efficacy, equity, ethics, and organizational implications. A survey was distributed to 110 APS specialists; 50 experts contributed responses (45.5% response rate; 66% clinicians, 18% laboratory personnel, 8% nurses, 8% administrative/other). For each domain, Z-scores were calculated to compare current diagnostic practice (AS IS) with a scenario integrating aPS/PT testing (TO BE). Correlation analyses explored relationships across domains. **Results**: Across all five domains, the TO BE scenario scored substantially higher than standard practice. The largest improvements were observed in perceived diagnostic efficacy (ΔZ = +2.65) and safety (ΔZ = +2.03), followed by equity (ΔZ = +2.25), ethical/social impact (ΔZ = +1.96), and organizational feasibility (ΔZ = +1.61). Perceived diagnostic effectiveness showed a strong positive correlation with both equity (r = 0.70, *p* < 0.001) and ethics (r = 0.67, *p* < 0.001). Participants consistently rated the assay as safe, clinically useful, equitable, and organizationally easy to introduce in routine laboratory workflows. **Conclusions**: Experts perceived the addition of aPS/PT testing as a meaningful enhancement to APS diagnostics, particularly for SLE patients who are seronegative on conventional assays. Its favorable profile across all HTA domains supports further evaluation in prospective cohorts and consideration for integration into future diagnostic algorithms.

## 1. Introduction

Antiphospholipid syndrome (APS) is an autoimmune, immune-mediated disorder characterized by recurrent vascular thromboses and/or pregnancy morbidity in the presence of persistent antiphospholipid antibodies (aPL) [1]. The current cornerstone of APS laboratory diagnosis relies on the detection of lupus anticoagulant (LA), anticardiolipin antibodies (aCL), and anti-β2-glycoprotein I antibodies (aβ2GPI) [2]. Despite their clinical utility, these assays do not fully capture the biological heterogeneity of aPL profiles, and clinicians frequently encounter patients in whom the clinical phenotype strongly suggests APS, but conventional laboratory tests remain persistently negative [3]. This situation is particularly common among patients with systemic lupus erythematosus (SLE), in whom the presence of thrombotic events, livedo reticularis, thrombocytopenia, or obstetric complications may raise high suspicion of APS despite negative testing for LA, aCL and aβ2GPI [3]. Such cases highlight the limitation of relying exclusively on the conventional aPL triad and underscore the need to explore additional biomarkers that might enhance diagnostic precision. Among emerging candidates, antibodies directed against prothrombin or the phosphatidylserine–prothrombin complex (aPS/PT) have received increasing attention [4,5,6,7,8,9,10]. Earlier work established that antibodies targeting prothrombin itself (aPT) differ immunologically and clinically from those directed against the phosphatidylserine-bound complex, with the latter more closely linked to LA activity and thrombotic manifestations [4,5,6,7,8,9,10]. Studies in both SLE and APS cohorts have demonstrated that aPS/PT antibodies may identify individuals at risk for thrombosis or pregnancy morbidity who would otherwise be missed by LA, aCL, and aβ2GPI assays [4,5,6,7,8,9,10]. Accordingly, several groups have proposed incorporating aPS/PT into diagnostic algorithms, particularly for evaluating patients at high clinical suspicion for APS or refining thrombotic risk stratification. Furthermore, ELISA harmonization and advances in assay technology, including chemiluminescent immunoassays, and particle-based multi-analyte platforms, have the potential to improve analytical performance, reproducibility, and accessibility of aPS/PT testing across laboratories, thereby increasing their feasibility as routine diagnostics [3].

This growing body of evidence has contributed to ongoing discussions about whether aPS/PT should be considered among “non-criteria” aPL of potential clinical relevance, and whether these markers, alone or in combination with existing tests, could strengthen future APS diagnostic strategies. The problem is not merely theoretical. Misclassification or delayed diagnosis of APS carries substantial clinical and societal consequences, including preventable stroke, recurrent miscarriages, avoidable anticoagulation, and inappropriate exposure to immunosuppressive or biologic therapies. Economic analyses of thrombotic disorders have consistently demonstrated that delayed or inaccurate diagnosis contributes disproportionately to long-term healthcare expenditure, reduced productivity, and loss of patient quality-adjusted life years [11,12,13,14]. In this context, refining APS diagnostics by identifying markers that address current gaps is not only a scientific objective but also a public health imperative [15]. However, despite the growing scientific interest in aPS/PT and the publication of several analytical validation studies, the broader implications of adopting this assay in real-world settings have received only partial attention. Laboratory diagnostics are embedded within complex healthcare systems in which decisions about test adoption must consider not only accuracy but also patient safety, equity of access, ethical acceptability, workflow integration, resource implications, and downstream clinical decision-making. Health Technology Assessment (HTA) provides a structured, multidimensional framework for evaluating diagnostic innovations by systematically examining their clinical, economic, ethical, organizational, and societal impact. HTA has been increasingly applied to infectious disease diagnostics, oncology molecular testing, and precision medicine platforms; yet, despite the centrality of autoantibody assays in APS, no formal HTA has previously been conducted for antiphospholipid antibody technologies [16]. In the context of APS, such an assessment is long overdue. The complexity of APS pathophysiology, the heterogeneity of clinical scenarios, and the reliance on laboratory confirmation make diagnostic strategies uniquely sensitive to system-level considerations. For instance, the organizational feasibility of integrating additional assays may differ substantially between specialized centers and peripheral laboratories. Ethical and equity implications are particularly relevant in rare diseases such as APS, where diagnostic uncertainty can exacerbate disparities and delay appropriate care [15]. Furthermore, the introduction of new biomarkers carries implications for cost-effectiveness, workforce training, quality assurance programs, and clinical guideline harmonization. To address these unmet needs, we conducted a comprehensive, multidomain HTA evaluating a commercially available aPS/PT assay within APS diagnostic pathways, with specific attention to SLE patients exhibiting high clinical suspicion but negative conventional aPL [17,18]. By systematically comparing the current diagnostic AS IS scenario with a hypothetical TO BE scenario incorporating aPS/PT, we sought to provide a rigorous assessment of the assay’s potential clinical value, feasibility, and broader system-level impact. This HTA represents, to our knowledge, one of the first structured attempts to apply a formal evaluation framework to APS diagnostics and contributes novel evidence to inform future laboratory practice, policy decisions, and evolving diagnostic algorithms.

## 2. Methods

### 2.1. Study Design

**Figure 1:**
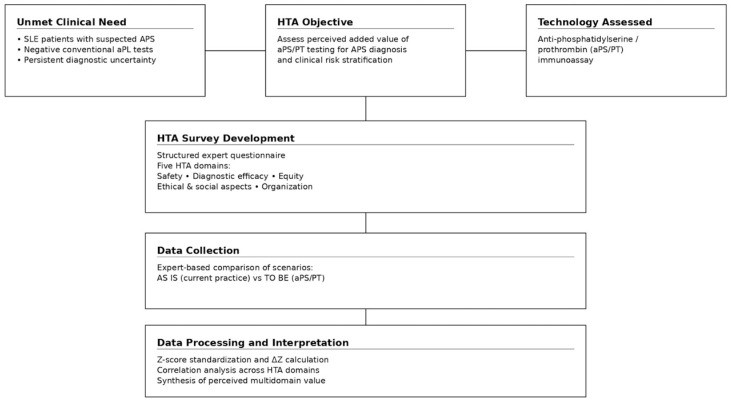
Workflow of the Health Technology Assessment (HTA) performed for the integration of anti-phosphatidylserine/prothrombin (aPS/PT) testing into APS diagnostics. The process included identification of the perceived diagnostic gap, selection of the technology to be evaluated, development of a multidisciplinary survey instrument based on five HTA domains, structured data collection comparing current practice (AS IS) with an aPS/PT-integrated scenario (TO BE), statistical processing using Z-score standardization and correlation analyses, and synthesis of results across domains.

We conducted a structured Health Technology Assessment (HTA) to evaluate the clinical, ethical, and organizational implications of introducing a commercially available aPS/PT immunoassay into the diagnostic workflow for patients with SLE and clinical suspicion of APS but negative conventional aPL. The study followed methodological principles aligned with the EUnetHTA Core Model and internationally recognized HTA frameworks for diagnostic technologies, with specific adaptation to laboratory medicine. The main steps of the workflow of the HTA performed for the integration of anti-phosphatidylserine/prothrombin (aPS/PT) testing into APS diagnostics are shown in Figure 1.

Two diagnostic scenarios were compared:

1. AS IS scenario: current standard of care using LA, aCL, and aβ2GPI as the sole laboratory tools.
2. TO BE scenario: integration of aPS/PT testing in addition to current standard assays.

The objective was to determine whether the TO BE scenario improved diagnostic processes across the five HTA domains.

This HTA was intentionally designed as a perception-based assessment relying on structured expert elicitation, an approach commonly used in early-phase evaluation of diagnostic technologies when empirical clinical or economic data are limited or unavailable.

### 2.2. Participants and Data Collection

A survey was distributed to experts affiliated with the APS Consortium of Piedmont and Aosta Valley, which includes specialists in clinical immunology, rheumatology, nephrology, hematology, laboratory medicine, nursing, and patient advocacy. The sampling frame consisted of 110 professionals. Respondents indicated their professional profile, years of experience, and completed all relevant domain-specific survey items. At the time of the study, none of the centers included had routinely implemented aPS/PT testing as part of their clinical practice.

### 2.3. Survey Instrument Development

A multidisciplinary team of clinicians, laboratory specialists, and methodologists developed the survey used for HTA data collection. Items were adapted from previously validated HTA instruments applied to immunological and serological diagnostics and refined through iterative consensus meetings. The survey was pilot-tested in a small group of experts (*n* = 8) for clarity, content validity, and internal consistency.

The final instrument comprised domain-specific items structured across five HTA dimensions: (A) Safety: perceived risks, tolerability, operator safety, diagnostic uncertainty. (B) Perceived efficacy: perceived diagnostic yield, contribution to risk stratification, impact on clinical decision-making. (C) Equity: accessibility across population subgroups and care settings, potential to reduce disparities. (D) Ethics and social implications: fairness, acceptability, patient-centeredness, societal value. (E) Organizational impact: perceived workflow integration, laboratory requirements, human resource allocation, and implementation complexity, explicitly excluding formal cost, reimbursement, or budget impact considerations.

Responses for each item were captured using a symmetric Likert scale (−3 to +3), with higher values indicating more favorable assessments. Importantly, respondents completed each item twice—once reflecting the AS IS scenario and once reflecting the TO BE scenario. Representative examples of survey items and the full Likert scale structure are provided in Supplementary Materials.

### 2.4. Standardization and Z-Score Calculation

To allow comparison across items with different scales and distributions, all responses were standardized using Z-scores:

$$Z=\frac{X-\mu }{\sigma }$$

where X was the raw score, μ the mean, and σ the standard deviation of the domain-specific distribution. For each domain, mean Z-scores were computed separately for the AS IS and TO BE scenarios. The primary outcome for each domain was the difference in mean Z-scores (ΔZ = TO BE − AS IS).

### 2.5. Statistical Analysis

Descriptive statistics were used to summarize participant characteristics. Domain-specific mean Z-scores were compared between the two scenarios using paired differences (ΔZ). Given the ordinal nature of the data, correlation analyses between domains were conducted using Spearman’s correlation coefficient (r), with significance preset at *p* < 0.05. Missing data were minimal (<5% per domain) and were handled by pairwise deletion. All analyses were performed using Python-based data processing (pandas 2.3.2) and standard statistical packages.

### 2.6. Visualization

Radar charts were constructed to provide a visual comparison of the AS IS and TO BE scenarios across the five HTA domains. Each axis represented a domain-level standardized Z-score, with outward extension indicating more favorable assessments.

## 3. Results

### 3.1. Participant Characteristics

A total of 50 experts completed the HTA survey. Respondents included 33 physicians (66%), 9 laboratory technicians (18%), 4 nurses (8%), 2 administrative professionals (4%), 1 biologist (2%), and 1 patient representative (2%). The median professional experience ranged from <5 years to >20 years, reflecting a heterogeneous and multidisciplinary panel representative of APS diagnostic pathways in the Piedmont and Aosta Valley regions. This expert composition reflects a high level of specialization in APS care and diagnostics, which should be considered when interpreting the findings.

### 3.2. Overall Comparison Between Scenarios

**Figure 2:**
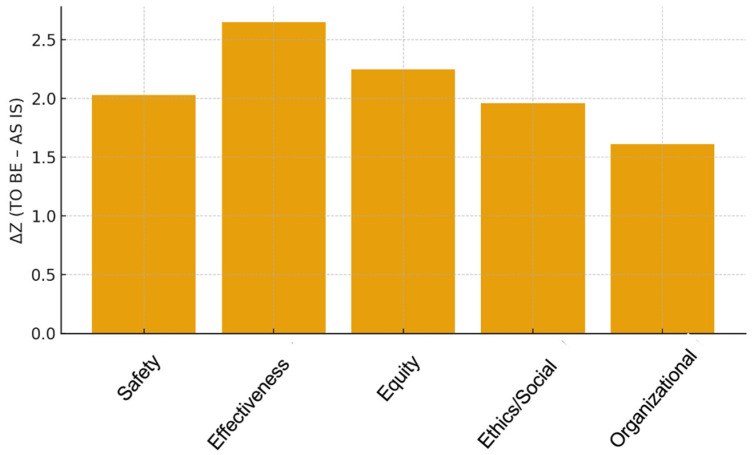
Improvements across the five Health Technology Assessment (HTA) domains following integration of aPS/PT testing (ΔZ = TO BE − AS IS).

Across all five HTA domains, safety, perceived efficacy, equity, ethics/social impact, and organizational implications, the TO BE scenario (integration of aPS/PT testing) demonstrated higher standardized Z-scores compared with the AS IS scenario (current diagnostic approach). These findings indicate a consistently more favorable expert evaluation for the implementation of aPS/PT testing (Figure 2).

### 3.3. Domain-Level Outcomes

#### 3.3.1. Safety

The introduction of aPS/PT testing was perceived to substantially enhance overall safety. The mean standardized score for the TO BE scenario was markedly higher than that of the AS IS scenario, yielding a ΔZ of +2.03. Improvements were attributed to better risk stratification, perceived reduction in diagnostic uncertainty, and enhanced patient and operator safety.

#### 3.3.2. Perceived Efficacy

The most pronounced improvement was observed in perceived diagnostic. Experts rated the TO BE scenario significantly higher, with a ΔZ of +2.65, reflecting strong confidence in the ability of aPS/PT testing to improve perceived diagnostic accuracy, refine risk assessment, and support earlier therapeutic decision-making in seronegative patients with suspected APS.

#### 3.3.3. Equity

The TO BE scenario was associated with improved perceptions of diagnostic equity, with a ΔZ of +2.25. Experts highlighted the potential of aPS/PT testing to reduce disparities in access to timely and reliable diagnosis, particularly for high-risk seronegative SLE patients who are currently underserved by conventional aPL assays.

#### 3.3.4. Ethics and Social Impact

Respondents identified ethical and social advantages linked to the implementation of aPS/PT testing, with a ΔZ of +1.96. The assay was perceived as contributing to more just allocation of diagnostic resources, reducing diagnostic ambiguity, and fostering more ethically grounded clinical decision-making.

#### 3.3.5. Organizational Implications

The perceived organizational impact was positive, with a ΔZ of +1.61. Respondents emphasized that the assay’s simple and rapid methodology would be readily integrated into existing laboratory workflows with minimal disruption, supporting its feasibility in routine practice.

**Figure 3:**
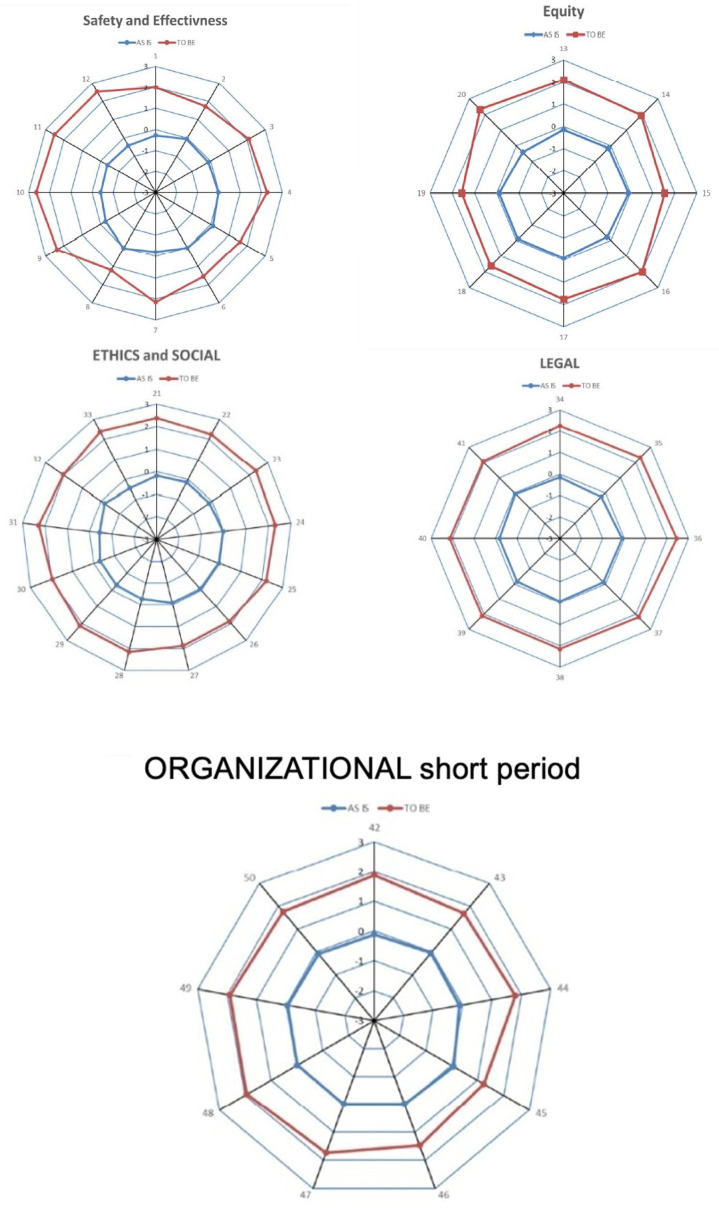
Radar charts comparing the AS IS (current practice) and TO BE (aPS/PT-integrated) diagnostic scenarios across the five HTA domains: safety, perceived efficacy, equity, ethics/social impact, and organizational implications. Higher scores indicate more favorable evaluations.

Figure 3 aimed to summarize the centrifugal improvement pattern associated with aPS/PT implementation.

### 3.4. Correlation Analyses

**Figure 4:**
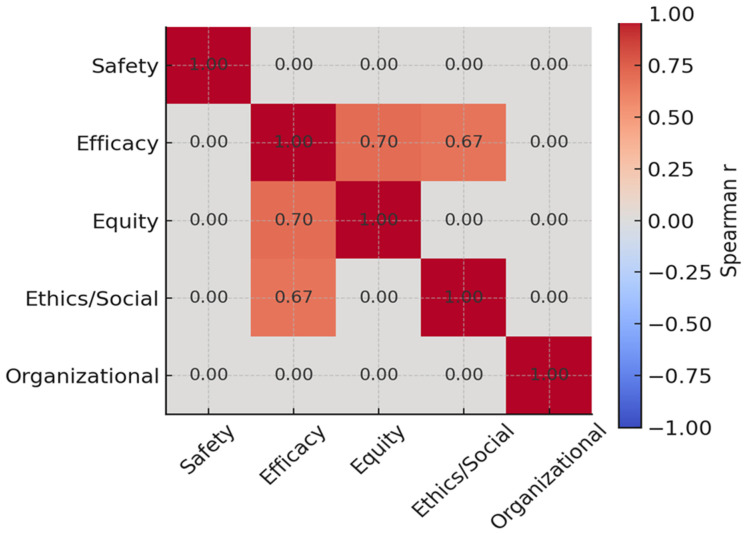
Spearman correlation matrix among the five HTA domains in the aPS/PT-integrated diagnostic scenario (TO BE).

Significant positive correlations were observed between perceived diagnostic efficacy and both equity (r = 0.70; *p* < 0.001) and ethics (r = 0.67; *p* < 0.001). These findings suggest that enhancements in diagnostic performance are closely linked to perceived improvements in fairness, societal value, and ethical practice (Figure 4).

### 3.5. Visual Summary

Radar-chart visualization demonstrated clear centrifugal expansion of domain scores in the TO BE scenario relative to the AS IS condition, graphically reinforcing the quantitative improvements across all HTA domains (Figure 2 Workflow of the Health Technology Assessment (HTA) performed for the integration of anti-phosphatidylserine/prothrombin (aPS/PT) testing into APS diagnostics).

## 4. Discussion

This multidomain Health Technology Assessment provides the first structured evaluation of integrating anti-phosphatidylserine/prothrombin testing into APS diagnostic pathways and reveals several consistent advantages across clinical, organizational, ethical, and societal domains. The findings demonstrate that experts perceived the addition of aPS/PT testing as a substantive improvement over current practice, with the TO BE scenario showing markedly higher Z-scores across all five HTA dimensions. These results underscore the potential of aPS/PT to address unmet diagnostic needs, particularly among SLE patients with a high clinical suspicion of APS who remain seronegative on conventional assays. The strongest perceived benefit emerged within the perceived diagnostic efficacy domain, where aPS/PT testing demonstrated a ΔZ of +2.65. This reflects growing recognition within the scientific community that aPS/PT antibodies capture immunologic specificities and pathogenic pathways not fully identified through LA, aCL, or aβ2GPI testing. Previous studies have shown that aPS/PT correlates with thrombotic risk, pregnancy morbidity, and LA activity, supporting its relevance as a complementary biomarker [4,5,6,7,8,9,10]. Importantly, aPS/PT antibodies have been detected in patients who would otherwise be considered “seronegative APS,” providing clinicians with actionable information in diagnostic dilemmas. Our findings extend this evidence base by demonstrating that experts do not view aPS/PT merely as an additional assay but as a test capable of meaningfully improving diagnostic yield and refining risk stratification. Safety and equity domains also demonstrated notable improvements in the TO BE scenario. The enhanced safety perception (ΔZ = +2.03) likely reflects two interrelated concepts: reduction in diagnostic uncertainty and improved identification of at-risk patients. Seronegative individuals who present with thrombotic or obstetric complications often undergo repeated cycles of testing, imaging, and empirical treatment, which can delay appropriate management and increase exposure to unnecessary therapies. By reducing diagnostic ambiguity, aPS/PT testing may support earlier and more tailored therapeutic decision-making, minimizing both under- and overtreatment. The improved equity scores (ΔZ = +2.25) highlight another important dimension: current APS diagnostics disproportionately disadvantage certain patient subgroups, e.g., women with SLE, younger patients, and those from settings with variable access to specialized laboratory assays. Respondents recognized that incorporating aPS/PT into routine workflows could reduce disparities in diagnostic accuracy, particularly for patients whose disease biology does not align with the conventional laboratory triad. Equity considerations are increasingly emphasized in rare disease policy frameworks, and our findings suggest that expanding aPL testing to include aPS/PT aligns with broader European and international commitments to promote equitable access to timely diagnosis.

The ethical and social domain also demonstrated meaningful improvement (ΔZ = +1.96). Ethical decision-making in rare autoimmune disorders often hinges on avoiding diagnostic delay, reducing uncertainty, and ensuring fairness in access to care [14]. Participants viewed aPS/PT testing as an ethically sound adjunct that enhances patient autonomy (through earlier and more confident diagnosis), supports shared decision-making, and improves justice by reducing structural barriers inherent in the current diagnostic approach. The strong correlations between perceived efficacy and both equity (r = 0.70) and ethics (r = 0.67) emphasize the interdependence of diagnostic accuracy, fairness, and ethical practice. When diagnostic performance improves, the downstream societal and ethical impact expands correspondingly. Organizational feasibility, with a ΔZ of +1.61, further supports the test’s translational potential. Respondents emphasized the assay’s compatibility with existing ELISA-based laboratory infrastructures, minimal additional training requirements, and rapid integration into current workflows. These features represent key facilitators of real-world adoption. Diagnostic innovations frequently fail to translate into practice due to organizational barriers rather than a lack of clinical utility. By identifying consistently favorable organizational evaluations, this HTA highlights the practical feasibility of introducing aPS/PT into diverse laboratory settings. Beyond the quantified HTA results, broader contextual factors support the relevance of integrating aPS/PT testing. The prevalence of APS remains relatively low, but its morbidity, including stroke and pregnancy loss, carries substantial individual and societal burden. Misdiagnosis or delayed diagnosis can lead to avoidable thrombotic events with long-term consequences. From a health-system perspective, improving diagnostic precision may contribute to cost savings by reducing unnecessary imaging, repeated testing, or inappropriate anticoagulation. Although this study did not incorporate a formal economic evaluation, the identified improvements across safety, perceived efficacy, and organizational domains indicate potential downstream health–economic benefits worthy of future investigation. Despite these strengths, several limitations merit consideration. First, this HTA reflects expert perception rather than measured clinical outcomes or formal cost-effectiveness analyses; therefore, the findings should be interpreted as hypothesis-generating and complementary to, rather than a substitute for, empirical validation studies. The improvements associated with the TO BE scenario represent anticipated benefits rather than measured effects. Prospective cohort studies and real-world implementation trials will be essential to validate these findings. Second, respondents were predominantly drawn from a regional APS network within a high-resource healthcare setting. While the multidisciplinary nature of the sample strengthens internal validity, the generalizability of the results may vary in international settings with different laboratory infrastructures, reimbursement models, or diagnostic pathways. The applicability of these findings must be interpreted in light of substantial variability across healthcare systems. Diagnostic adoption is strongly influenced by contextual factors, including laboratory infrastructure, workforce expertise, reimbursement mechanisms, and national or regional policy frameworks. In high-resource settings with established autoimmune diagnostic laboratories, integration of aPS/PT testing may be facilitated by existing platforms and centralized referral pathways. In contrast, in low-resource or decentralized settings, limited access to specialized assays, constrained laboratory capacity, and competing diagnostic priorities may reduce feasibility or delay implementation. Reimbursement models represent another critical source of variability. In publicly funded health systems with bundled laboratory reimbursement, the addition of new assays may require formal health–economic justification or national-level approval, whereas in mixed or private reimbursement models, adoption may occur more rapidly but with greater variability in access. Consequently, perceptions of organizational feasibility and value captured in this study may not translate uniformly across jurisdictions. For these reasons, the results of this perception-based HTA should not be considered globally generalizable. Rather, they reflect the perspectives of a specialized regional network operating within a high-resource European healthcare context. Future research should extend this framework to diverse international settings, including non-specialized laboratories and health systems with different resource constraints and reimbursement structures, to better define the scalability and transferability of aPS/PT testing.

Also, the sample size was relatively modest and restricted to a single Italian macro-region. Participants were recruited from a dedicated APS consortium, introducing potential selection bias, as respondents may have greater awareness of or confidence in the clinical utility of aPS/PT testing. As a result, the external validity of the findings may be limited, and perceptions of feasibility or value may differ in healthcare systems with lower levels of APS specialization, different laboratory infrastructures, or alternative reimbursement models. Consequently, these results should be interpreted as context-specific and hypothesis-generating rather than universally generalizable. Third, although aPS/PT testing has a strong biological and clinical rationale, the marker is not yet incorporated into APS classification criteria, and consensus regarding its role in disease definition is still evolving. Its integration into diagnostic workflows must therefore be considered complementary rather than substitutive. Nevertheless, the present HTA represents a significant contribution to APS diagnostics. By applying a structured, multidimensional framework, this study situates aPS/PT testing within a broader health-system context, moving beyond analytical validity to assess feasibility, ethical alignment, and organizational readiness. These findings suggest that aPS/PT testing has the potential to fill critical gaps in current APS diagnostics and could meaningfully improve diagnostic pathways for seronegative but clinically high-risk patients. Also, although organizational feasibility was rated favorably, this domain reflects operational and workflow considerations rather than economic value. Formal cost-effectiveness and budget impact analyses remain essential and were beyond the scope of this perception-based HTA. Additionally, collaboration between clinical immunology, rheumatology, laboratory medicine, and regulatory bodies will be essential to ensure that promising biomarkers such as aPS/PT are appropriately integrated into practice and, when evidence warrants, into classification criteria [19,20].

Although this HTA is perception-based, the perceived improvements across domains are supported by plausible clinical and organizational mechanisms. By reducing diagnostic uncertainty, particularly in patients negative for criteria aPL with high clinical suspicion of APS, the integration of aPS/PT testing may enable earlier diagnostic clarification and more consistent risk stratification. This, in turn, could support timelier and more tailored therapeutic decisions, including appropriate initiation or avoidance of long-term anticoagulation and improved counseling in high-risk settings such as pregnancy. At the healthcare system level, enhanced diagnostic confidence may reduce repeated testing, fragmented care pathways, and unnecessary investigations, thereby improving workflow efficiency and alignment between laboratory diagnostics and clinical decision-making. While these anticipated effects require prospective validation, they provide a coherent framework linking expert-perceived value to potential measurable improvements in patient management and care delivery.

Future studies should extend this HTA framework to multicenter and international cohorts, including non-specialized laboratories and healthcare systems with varying resource availability, to better assess the generalizability and scalability of aPS/PT testing. Ultimately, improving APS diagnostics is not merely a laboratory challenge but a systems-level endeavor, and this HTA provides a foundation for evidence-based decision-making to support more equitable, ethical, and effective care for patients with suspected APS.

## Data Availability

The original contributions presented in this study are included in the article/Supplementary Material. Further inquiries can be directed to the corresponding author.

## Supplementary Materials

The following supporting information can be downloaded at: https://www.mdpi.com/article/10.3390/diagnostics16030434/s1, Survey Structure and Example Survey Items by HTA Domain.
