# Supplementary material for: Integrating Anti-Phosphatidylserine/Prothrombin Antibodies Testing into Antiphospholipid Syndrome Diagnostics: A Multidomain, Expert Perception-Based Health Technology Assessment
Source: Diagnostics (Basel). 2026 Feb 1;16(3):434. doi: 10.3390/diagnostics16030434 (PMC12896444; doi:10.3390/diagnostics16030434)
Supplement: Supplementary file 1 [file diagnostics-16-00434-s001.zip › diagnostics-4042694-supplementary.pdf]

Survey Structure

The Health Technology Assessment (HTA) survey was designed to capture expert perceptions across five predefined domains: safety, diagnostic efficacy, equity, ethical and social implications, and organizational impact. For each item, respondents were asked to provide two ratings: one reflecting the current diagnostic approach (AS IS) and one reflecting a hypothetical scenario including aPS/PT testing (TO BE).

Likert Scale

All items were scored using a symmetric 7-point Likert scale ranging from –3 to +3, where:

–3 = strongly unfavorable

–2 = unfavorable

–1 = slightly unfavorable

0 = neutral

+1 = slightly favorable

+2 = favorable

+3 = strongly favorable

Higher scores indicated a more favorable perception of the diagnostic approach being evaluated.

Example Survey Items by HTA Domain

B. Diagnostic Efficacy

How effectively does the diagnostic strategy identify patients at risk of APS-related complications?

To what extent does the diagnostic approach support clinical decision-making in seronegative (negative for criteria aPL) patients?
